# Supplementary material for: A systematic review and meta-analysis of indoor bioaerosols in hospitals: The influence of heating, ventilation, and air conditioning
Source: PLoS One. 2021 Dec 23;16(12):e0259996. doi: 10.1371/journal.pone.0259996 (PMC8699671; doi:10.1371/journal.pone.0259996)
Supplement: S1 File — (DOCX) [file pone.0259996.s001.docx]

**Embase Session Results (31 Dec 2020)**

No. Query Results

#85 #11 AND #22 AND #46 AND #82 AND [2000-2020]/py AND [english]/lim 9978

#84 #11 AND #22 AND #46 AND #82 AND [2000-2020]/py 10405

#83 #11 AND #22 AND #46 AND #82 12067

#82 #47 OR #48 OR #49 OR #50 OR #51 OR #52 OR #53 OR #54 OR #55 OR #56 OR #57 OR #58 OR #59 OR #60 OR #61 OR #62 OR #63 OR #64 OR #65 OR #66 OR #67 OR #68 OR #69 OR #70 OR #71 OR #72 OR #73 OR #74 OR #75 OR #76 OR #77 OR #78 OR #79 OR #80 OR #81 10471127

#81 'cfu'/exp 42702

#80 'cfu*':ti,ab,kw 60330

#79 'chlamydia*':ti,ab,kw 35443

#78 'chlamydia'/exp 34563

#77 'mycoplasma*':ti,ab,kw 27804

#76 'mycoplasma'/exp 27367

#75 'atmosphere qualit*':ti,ab,kw 16

#74 'particulate*':ti,ab,kw 70274

#73 'microorganism*':ti,ab,kw 140944

#72 'microorganism'/exp 128994

#71 'respiratory tract diseases'/exp 2762566

#70 'coronavirus*':ti,ab,kw 40756

#69 'coronavirus'/exp 43141

#68 'germ*':ti,ab,kw 422239

#67 'germ'/exp 34

#66 'air qualit*':ti,ab,kw OR 'aqi':ti,ab,kw OR 'iqa':ti,ab,kw 20501

#65 'air quality'/exp 31852

#64 'pm2.5':ti,ab,kw 5706

#63 'pm2.5'/exp 572

#62 'dust*':ti,ab,kw 66692

#61 'dust'/exp 36451

#60 'eubacteria':ti,ab,kw OR 'eubacterium':ti,ab,kw 5484

#59 'mold*':ti,ab,kw 30241

#58 'mold'/exp 13431

#57 'bacteria'/exp 1791469

#56 'fungi'/exp 549804

#55 'virus'/exp 1131458

#54 'inhalation exposure':ti,ab,kw 4241

#53 'virolog*':ti,ab,kw 59014

#52 'air microbiology'/exp 471204

#51 'inhalation exposure'/exp 650009

#50 'bacter*':ti,ab,kw OR 'virus*':ti,ab,kw OR 'viral':ti,ab,kw OR 'fung*':ti,ab,kw OR 'mycos*':ti,ab,kw OR 'micro*':ti,ab,kw OR 'micro-org*':ti,ab,kw OR 'bacteriol*':ti,ab,kw OR 'mycolog*':ti,ab,kw 5011838

#49 'virus diseases'/exp 1289082

#48 'mycoses'/exp 208478

#47 'bacterial infections'/exp 1049694

#46 #23 OR #24 OR #25 OR #26 OR #27 OR #28 OR #29 OR #30 OR #31 OR #32 OR #33 OR #34 OR #35 OR #36 OR #37 OR #38 OR #39 OR #40 OR #41 OR #42 OR #43 OR #44 OR #45 2179945

#45 'climate control*':ti,ab,kw 515

#44 'air delivery system*':ti,ab,kw 14

#43 'air revitalization':ti,ab,kw 21

#42 'humidification system*':ti,ab,kw 115

#41 'ventilation system*':ti,ab,kw 2035

#40 'cooling system*':ti,ab,kw 1431

#39 'heating system*':ti,ab,kw 1156

#38 'controlled':ti,ab,kw AND 'environment*':ti,ab,kw 48549

#37 'hvac':ti,ab,kw 555

#36 'ventilation*':ti,ab,kw 187507

#35 'ventilation'/exp 24050

#34 'fan*':ti,ab,kw 38604

#33 'fan'/exp 21

#32 'radiant':ti,ab,kw AND 'cool*':ti,ab,kw 232

#31 'air':ti,ab,kw AND 'condition*':ti,ab,kw 58867

#30 'air conditioning'/exp 24045

#29 'filtration*':ti,ab,kw 159597

#28 'filtration'/exp 78784

#27 'recirculation*':ti,ab,kw 9915

#26 'recirculation'/exp 86

#25 'mechanical ventilation*':ti,ab,kw OR 'mechannical system*':ti,ab,kw 72693

#24 'health facilit*':ti,ab,kw 18716

#23 'health facilities'/exp 1679940

#22 #12 OR #13 OR #14 OR #15 OR #16 OR #17 OR #18 OR #19 OR #20 OR #21 7947484

#21 'inpatient facilit*':ti,ab,kw 1128

#20 'medical establishment*':ti,ab,kw 658

#19 'outpatient service*':ti,ab,kw OR 'outpatient care*':ti,ab,kw OR 'health service*':ti,ab,kw 163156

#18 'outpatient service'/exp 37656

#17 'intensive care unit*':ti,ab,kw OR 'icu*':ti,ab,kw OR 'picu*':ti,ab,kw 244876

#16 'intensive care unit'/exp 203360

#15 'inpatient ward*':ti,ab,kw OR 'sickroom*':ti,ab,kw OR 'infirmar*':ti,ab,kw OR 'ambulatory care*':ti,ab,kw 19535

#14 'ambulatory care'/exp 51190

#13 'hospital'/exp 1197785

#12 'hospital':ti,ab,kw OR 'hospitals':ti,ab,kw OR 'ward':ti,ab,kw OR 'wards':ti,ab,kw OR 'unit':ti,ab,kw OR 'units':ti,ab,kw OR 'clinic':ti,ab,kw OR 'clinics':ti,ab,kw OR 'clinical':ti,ab,kw OR 'operating room*':ti,ab,kw OR 'operating theat*':ti,ab,kw OR 'healthcare':ti,ab,kw 7541422

#11 #1 OR #2 OR #3 OR #4 OR #5 OR #6 OR #7 OR #8 OR #9 OR #10 301439

#10 'aerocolloid':ti,ab,kw 2

#9 'gasoloid':ti,ab,kw 1

#8 'atomosphere*':ti,ab,kw 15

#7 'air'/exp 51739

#6 'airborne':ti,ab,kw OR 'air-borne':ti,ab,kw 29780

#5 'air pollution*':ti,ab,kw OR 'air pollutant*':ti,ab,kw OR 'air contaminat*':ti,ab,kw 47293

#4 'air pollutants'/exp 81511

#3 'air pollution'/exp 167900

#2 'bioaerosol*':ti,ab,kw OR 'bio-aerosol*':ti,ab,kw OR 'aerosol*':ti,ab,kw 69219

#1 'aerosols'/exp 60312

**Cochrane Library Session Results (31 Dec 2020)**

ID Search

#1 MeSH descriptor: [Aerosols] explode all trees

#2 MeSH descriptor: [Air Pollution] explode all trees

#3 MeSH descriptor: [Air] explode all trees

#4 “bioaerosol*”:ti,ab,kw OR “bio-aerosol*”:ti,ab,kw OR “aerosol*”:ti,ab,kw

#5 “air pollution*”:ti,ab,kw OR “air pollutant*”:ti,ab,kw OR “air contaminat*”:ti,ab,kw

#6 “airborne”:ti,ab,kw OR “air-borne”:ti,ab,kw

#7 “atomosphere*”:ti,ab,kw

#8 “gasoloid”:ti,ab,kw

#9 “aerocolloid”:ti,ab,kw

#10 #1 or #2 #3 or #4 or #5 or #6 or #7 or #8 or #9

#11 MeSH descriptor: [Hospitals] explode all trees

#12 MeSH descriptor: [Intensive Care Units] explode all trees

#13 MeSH descriptor: [Ambulatory Care] explode all trees

#14 “hospital”:ti,ab,kw OR “hospitals”:ti,ab,kw OR “ward”:ti,ab,kw OR “wards”:ti,ab,kw OR “unit”:ti,ab,kw OR “units”:ti,ab,kw OR “clinic”:ti,ab,kw OR “clinics”:ti,ab,kw OR “clinical”:ti,ab,kw OR “operating room*”:ti,ab,kw OR “operating theat*”:ti,ab,kw OR “healthcare”:ti,ab,kw

#15 “infirmar*”:ti,ab,kw

#16 “inpatient ward*”:ti,ab,kw OR “sickroom*”:ti,ab,kw

#17 “intensive care unit*”:ti,ab,kw OR “icu*”:ti,ab,kw OR “picu*”:ti,ab,kw

#18 “outpatient service*”:ti,ab,kw OR “outpatient care*”:ti,ab,kw OR “health service*”:ti,ab,kw

#19 “medical establishment*”:ti,ab,kw

#20 “inpatient facilit*”:ti,ab,kw

#21 #11 or #12 OR #13 OR #14 OR #15 OR #16 OR #17 OR #18 OR #19 OR #20

#22 MeSH descriptor: [Health Facilities] explode all trees

#23 MeSH descriptor: [Filtration] explode all trees

#24 MeSH descriptor: [Air Conditioning] explode all trees

#25 MeSH descriptor: [Ventilation] explode all trees

#26 “health facilit*”:ti,ab,kw

#27 “mechanical ventilation*”:ti,ab,kw OR “mechannical system*”:ti,ab,kw

#28 “recirculation*”:ti,ab,kw

#29 “filtration*”:ti,ab,kw

#30 “air”:ti,ab,kw AND “condition*”:ti,ab,kw

#31 “radiant”:ti,ab,kw AND “cool*”:ti,ab,kw

#32 “fan*”:ti,ab,kw

#33 “ventilation”:ti,ab,kw

#34 “hvac”:ti,ab,kw

#35 “controlled”:ti,ab,kw AND “environment*”:ti,ab,kw

#36 “heating system*”:ti,ab,kw

#37 “cooling system*”:ti,ab,kw

#38 “ventilation system*”:ti,ab,kw

#39 “humidification system*”:ti,ab,kw

#40 “air revitalization”:ti,ab,kw

#41 “air delivery system*”:ti,ab,kw

#42 “climate control*”:ti,ab,kw

#43 #22 or #23 OR #24 OR #25 OR #26 OR #27 OR #28 OR #29 OR #30 OR #31 OR #32 OR #33 OR #34 OR #35 OR #36 OR #37 OR #38 OR #39 OR #40 OR #41 OR #42

#44 MeSH descriptor: [Bacterial Infections] explode all trees

#45 MeSH descriptor: [Bacterial Infections and Mycoses] explode all trees

#46 MeSH descriptor: [Virus Diseases] explode all trees

#47 MeSH descriptor: [Inhalation Exposure] explode all trees

#48 MeSH descriptor: [Air Microbiology] explode all trees

#49 MeSH descriptor: [Viruses] explode all trees

#50 MeSH descriptor: [Fungi] explode all trees

#51 MeSH descriptor: [Bacteria] explode all trees

#52 MeSH descriptor: [Dust] explode all trees

#53 MeSH descriptor: [Coronavirus] explode all trees

#54 MeSH descriptor: [Respiratory Tract Diseases] explode all trees

#55 MeSH descriptor: [Organism Forms] explode all trees

#56 MeSH descriptor: [Mycoplasma] explode all trees

#57 MeSH descriptor: [Chlamydia] explode all trees

#58 “bacter*”:ti,ab,kw OR “virus*”:ti,ab,kw OR “viral”:ti,ab,kw OR “fung*”:ti,ab,kw or “mycos*”:ti,ab,kw OR “micro*”:ti,ab,kw OR “micro-org*”:ti,ab,kw OR “bacteriol*”:ti,ab,kw OR “mycolog*”:ti,ab,kw OR “virolog*”:ti,ab,kw

#59 “inhalation exposure”:ti,ab,kw

#60 “mold*”:ti,ab,kw

#61 “eubacteria”:ti,ab,kw OR “eubacterium”:ti,ab,kw

#62 “dust*”:ti,ab,kw

#63 “pm2.5”:ti,ab,kw

#64 “air qualit*”:ti,ab,kw OR “aqi”:ti,ab,kw OR “iqa”:ti,ab,kw

#65 “germ*”:ti,ab,kw

#66 “coronavirus*”:ti,ab,kw

#67 “microorganism*”:ti,ab,kw

#68 “particulate*”:ti,ab,kw

#69 “atmosphere qualit*”:ti,ab,kw

#70 “mycoplasma*”:ti,ab,kw

#71 “chlamydia*”:ti,ab,kw

#72 "cfu":ti,ab,kw

#73 #44 or #45 or #46 or #47 OR #48 OR #49 OR #50 OR #51 OR #52 OR #53 OR #54 OR #55 OR #56 OR #57 OR #58 OR #59 OR #60 OR #61 OR #62 OR #63 OR #64 OR #65 OR #66 OR #67 OR #68 OR #69 OR #70 OR #71 or #72

#74 #10 and #21 and #43 and #73

**Pubmed Session Results (31 Dec 2020)**

Search: (((((((((("Aerosols"[mesh]) OR ("Air Pollution"[mesh])) OR ("air"[mesh])) OR ("bioaerosol*"[title/abstract] OR "bio-aerosol*"[title/abstract] OR "aerosol*"[title/abstract])) OR ("air pollution*"[title/abstract] OR "air pollutant*"[title/abstract] OR "air contaminat*"[title/abstract])) OR ("airborne"[title/abstract] OR "air-borne"[title/abstract])) OR ("atomosphere*"[title/abstract])) OR ("aerocolloid"[title/abstract])) AND (((((((((("hospitals"[mesh]) OR ("intensive care units"[mesh])) OR ("ambulatory care"[mesh])) OR ("hospital"[title/abstract] OR "hospitals"[title/abstract] OR "ward"[title/abstract] OR "wards"[title/abstract] OR "unit"[title/abstract] OR "units"[title/abstract] OR "clinic"[title/abstract] OR "clinics"[title/abstract] OR "clinical"[title/abstract] OR "operating room*"[title/abstract] OR "operating theat*"[title/abstract] OR "healthcare"[title/abstract])) OR ("infirmar*"[title/abstract])) OR ("inpatient ward*"[title/abstract] OR "sickroom*"[title/abstract])) OR ("intensive care unit*"[title/abstract] OR "icu*"[title/abstract] OR "picu*"[title/abstract])) OR ("outpatient service*"[title/abstract] OR "outpatient care*"[title/abstract] OR "health service*"[title/abstract])) OR ("medical establishment*"[title/abstract])) OR ("inpatient facilit*"[title/abstract]))) AND ((((((((((((((((((((("Health Facilities"[mesh]) OR ("Filtration"[mesh])) OR ("Air Conditioning"[mesh])) OR ("Ventilation"[mesh])) OR ("health facilit*"[title/abstract])) OR ("mechanical ventilation*"[title/abstract])) OR ("recirculation*"[title/abstract])) OR ("filtration*"[title/abstract])) OR ("air"[title/abstract] AND "condition*"[title/abstract])) OR ("radiant"[title/abstract] AND "cool*"[title/abstract])) OR ("fan*"[title/abstract])) OR ("ventilation"[title/abstract])) OR ("hvac"[title/abstract])) OR ("controlled"[title/abstract] AND "environment*"[title/abstract])) OR ("heating system*"[title/abstract])) OR ("cooling system*"[title/abstract])) OR ("ventilation system*"[title/abstract])) OR ("humidification system*"[title/abstract])) OR ("air revitalization"[title/abstract])) OR ("air delivery system*"[title/abstract])) OR ("climate control*"[title/abstract]))) AND ((((((((((((((((((((((((((((("cfu*"[title/abstract]) OR ("chlamydia*"[title/abstract])) OR ("mycoplasma*"[title/abstract])) OR ("atmosphere qualit*"[title/abstract])) OR ("particulate*"[title/abstract])) OR ("microorganism*"[title/abstract])) OR ("coronavirus*"[title/abstract])) OR ("germ*"[title/abstract])) OR ("air qualit*"[title/abstract] OR "aqi"[title/abstract] OR "iqa"[title/abstract])) OR ("pm2.5"[title/abstract])) OR ("dust*"[title/abstract])) OR ("eubacteria"[title/abstract] OR "eubacterium"[title/abstract])) OR ("mold*"[title/abstract])) OR ("inhalation exposure"[title/abstract])) OR ("bacter*"[title/abstract] OR "virus*"[title/abstract] OR "viral"[title/abstract] OR "fung*"[title/abstract] or "mycos*"[title/abstract] OR "micro*"[title/abstract] OR "micro-org*"[title/abstract] OR "bacteriol*"[title/abstract] OR "mycolog*"[title/abstract] OR "virolog*"[title/abstract])) OR ("Chlamydia"[mesh])) OR ("Mycoplasma"[mesh])) OR ("Organism Forms"[mesh])) OR ("Respiratory Tract Diseases"[mesh])) OR ("Coronavirus"[mesh])) OR ("Dust"[mesh])) OR ("Bacteria"[mesh])) OR ("Fungi"[mesh])) OR ("Viruses"[mesh])) OR ("Air Microbiology"[mesh])) OR ("Inhalation Exposure"[mesh])) OR ("Virus Diseases"[mesh])) OR ("Bacterial Infections and Mycoses"[mesh])) OR ("Bacterial Infections"[mesh])) Sort by: Most Recent

**Web of Science Session Results (31 Dec 2020)**

# 42

14627

#41

AND

#24

AND

#13

AND

#6

# 41

21,759,767

#40

OR

#39

OR

#38

OR

#37

OR

#36

OR

#35

OR

#34

OR

#33

OR

#32

OR

#31

OR

#30

OR

#29

OR

#28

OR

#27

OR

#26

OR

#25

# 40

8,905,767

ti=("cfu"

OR “inhalation

exposure”

OR

“mold*”

OR

“eubacteria”

OR

“eubacterium”

OR

“dust*”

OR

“pm2.5”

OR

“air

qualit*”

OR

“aqi”

OR

“iqa”

OR

“germ*”

OR

“coronavirus*”

OR

“microorganism*”

OR

“particulate*”

OR

“atmosphere

qualit*”

OR

“mycoplasma*”

OR

“chlamydia*”)

OR

ab=("cfu"

OR

"inhalation

exposure”

OR

“mold*”

OR

“eubacteria”

OR

“eubacterium”

OR

“dust*”

OR

“pm2.5”

OR

“air

qualit*”

OR

“aqi”

OR

“iqa”

OR

“germ*”

OR

“coronavirus*”

OR

“microorganism*”

OR

“particulate*”

OR

“atmosphere qualit*”

OR

“mycoplasma*”

OR

“chlamydia*”)

OR

kp=("cfu"

OR

“inhalation

exposure”

OR

“mold*”

OR

“eubacteria”

OR

“eubacterium”

OR

“dust*”

OR

“pm2.5”

OR

“air

qualit*”

OR

“aqi”

OR

“iqa”

OR

“germ*”

OR

“coronavirus*”

OR

“microorganism*”

OR

“particulate*”

OR

“atmosphere

qualit*”

OR

“mycoplasma*”

OR

“chlamydia*”)

# 39

18,126,469

ti=(“bacter*”

OR

“virus*”

OR

“viral”

OR

“fung*”

OR

“mycos*”

OR

“micro*”

OR

“micro-org*”

OR

“bacteriol*”

OR

“mycolog*”

OR

“virolog*”)

OR

ab=(“bacter*”

OR

“virus*”

OR

“viral”

OR

“fung*”

OR

“mycos*”

OR

“micro*”

OR

“micro-org*”

OR

“bacteriol*”

OR

“mycolog*”

OR

“virolog*”)

OR

kp=(“bacter*”

OR

“virus*”

OR

“viral”

OR

“fung*”

OR

“mycos*”

OR

“micro*”

OR

“micro-org*”

OR

“bacteriol*”

OR

“mycolog*”

OR

“virolog*”)

# 38

55,725

ts="Chlamydia"

# 37

50,473

ts="Mycoplasma"

# 36

68

ts="Organism Forms"

# 35

24,282

ts="Respiratory Tract Diseases"

# 34

86,330

ts="Coronavirus"

# 33

962,518

ts="dust"

# 32

3,312,555

ts="Bacteria"

# 31

1,147,468

ts="Fungi"

# 30

2,016,284

ts="Viruses"

# 29

8,130

ts="Air Microbiology"

# 28

14,619

ts="Inhalation Exposure"

# 27

67,900

ts="Virus Diseases"

# 26

57

ts="Bacterial Infections

and

Mycoses"

# 25

418,078

ts="Bacterial Infections"

# 24

3,533,338

#23

OR

#22

OR

#21

OR

#20

OR

#19

OR

#18

OR

#17

OR

#16

OR

#15

OR

#14

# 23

255,984

ti=(“heating system*”

OR

“cooling system*”

OR

“ventilation system*”

OR

“humidification system*”

OR

“air revitalization”

OR

“air delivery system*”

OR

“climate control*”)

OR

ab=(“heating system*”

OR

“cooling system*”

OR

“ventilation system*”

OR

“humidification system*”

OR

“air

revitalization”

OR

“air delivery system*”

OR

“climate control*”)

OR

kp=(“heating system*”

OR

“cooling system*”

OR

“ventilation system*”

OR

“humidification system*”

OR

“air revitalization”

OR

“air delivery system*”

OR

“climate control*”)

# 22

259,577

ti=(“controlled”

AND

“environment*”)

OR

ab=(“controlled”

AND

“environment*”

)

OR

kp=(“controlled”

AND

“environment*”)

# 21

1,683,812

ti=(“fan*”

OR

"ventilation"

OR

“hvac”

)

OR

ab=(“fan*”

OR

"ventilation"

OR

“hvac”

)

OR

kp=(“fan*”

OR

"ventilation"

OR

“hvac”

)

# 20

5,998

ti=(“radiant”

AND

“cool*”)

OR

ab=(“radiant”

AND

“cool*”)

OR

kp=(“radiant”

AND

“cool*”)

# 19

950,346

ti=(“air”

AND

“condition*”)

OR

ab=(“air”

AND

“condition*”)

OR

kp=(“air”

AND

“condition*”)

# 18

712,452

ti=(“health facilit*”

OR

“mechanical ventilation*”

OR

“mechannical system*”

OR

“recirculation*”

OR

“filtration*”

)

OR

ab=(“health facilit*”

OR

“mechanical ventilation*”

OR

“mechannical system*”

OR

“recirculation*”

OR

“filtration*”

)

OR

kp=(“health facilit*”

OR

“mechanical ventilation*”

OR

“mechannical system*”

OR

“recirculation*”

OR

“filtration*”

)

# 17

656,935

ts="Ventilation"

# 16

184,858

ts="Air Conditioning"

# 15

512,645

ts="Filtration"

# 14

29,739

ts="Health Facilities"

# 13

22,552,116

#12

OR

#11

OR

#10

OR

#9

OR

#8

OR

#7

# 12

809,944

ti=(

“outpatient service*”

OR

“outpatient care*”

OR

“health service*”

OR

“medical establishment*”

OR

“inpatient facilit*”)

OR

ab=(

“outpatient

service*”

OR

“outpatient

care*”

OR

“health service*”

OR

“medical establishment*”

OR

“inpatient facilit*”)

OR

kp=(

“outpatient

service*”

OR

“outpatient

care*”

OR

“health service*”

OR

“medical

establishment*”

OR

“inpatient facilit*”)

# 11

256,714

ti=(“infirmar*”

OR

“inpatient ward*”

OR

“sickroom*”

OR

“intensive care unit*”

OR

“icu*”

OR

“picu*”)

OR

ab=(“infirmar*”

OR

“inpatient

ward*”

OR

“sickroom*”

OR

“intensive care unit*”

OR

“icu*”

OR

“picu*”)

OR

kp=(“infirmar*”

OR

“inpatient

ward*”

OR

“sickroom*”

OR

“intensive care unit*”

OR

“icu*”

OR

“picu*”)

# 10

22,112,397

ti=(“hospital”

OR

“hospitals”

OR

“ward”

OR

“wards”

OR

“unit”

OR

“units”

OR

“clinic”

OR

“clinics”

OR

“clinical”

OR

“operating room*”

OR

“operating theat*”

OR

“healthcare”)

OR

ab=(“hospital”

OR

“hospitals”

OR

“ward”

OR

“wards”

OR

“unit”

OR

“units”

OR

“clinic”

OR

“clinics”

OR

“clinical”

OR

“operating room*”

OR

“operating

theat*”

OR

“healthcare”)

OR

kp=(“hospital”

OR

“hospitals”

OR

“ward”

OR

“wards”

OR

“unit”

OR

“units”

OR

“clinic”

OR

“clinics”

OR

“clinical”

OR

“operating room*”

OR

“operating theat*”

OR

“healthcare”)

# 9

154,434

ts="intensive

care

unit"

# 8

568,258

ts="hospitals"

# 7

71,567

ts="Ambulatory Care"

# 6

6,129,283

#5

OR

#4

OR

#3

OR

#2

OR

#1

# 5

149,601

ti=(“airborne”

OR

“air-borne”

OR

“atomosphere*”

OR

“gasoloid”

OR

“aerocolloid”)

OR

ab=(“airborne”

OR

“air-borne”

OR

“atomosphere*”

OR

“gasoloid”

OR

“aerocolloid”)

OR

kp=(“airborne”

OR

“air-borne”

OR

“atomosphere*”

OR

“gasoloid”

OR

“aerocolloid”)

# 4

545,357

ti=(“bioaerosol*”

OR

“bio-aerosol*”

OR

“aerosol*”

OR

“air pollution*”

OR

“air pollutant*”

OR

“air contaminat*”)

OR

ab=(“bioaerosol*”

OR

“bio-aerosol*”

OR

“aerosol*”

OR

“air pollution*”

OR

“air pollutant*”

OR

“air contaminat*”)

OR

kp=(“bioaerosol*”

OR

“bio-aerosol*”

OR

“aerosol*”

OR

“air pollution*”

OR

“air pollutant*”

OR

“air contaminat*”)

# 3

5,854,527

ts="air"

# 2

255,189

ts="air pollution"

# 1

137,681

ts="aerosols"
