# Supplementary material for: A systematic review and meta-analysis of indoor bioaerosols in hospitals: The influence of heating, ventilation, and air conditioning
Source: PLoS One. 2021 Dec 23;16(12):e0259996. doi: 10.1371/journal.pone.0259996 (PMC8699671; doi:10.1371/journal.pone.0259996)
Supplement: S1 Appendix — (DOCX) [file pone.0259996.s002.docx]

Appendix_3

Quality Assessment Forms

|  |  |  |  |
| --- | --- | --- | --- |
| **Item** | **Yes** | **No** | **Unclear** |
| 1) Define the source of information (survey, record review) |  |  |  |
| 2) List inclusion and exclusion criteria for exposed and unexposed subjects (cases and controls) or refer to previous publications |  |  |  |
| 3) Indicate time period used for identifying patients |  |  |  |
| 4) Indicate whether or not subjects were consecutive if not population-based |  |  |  |
| 5) Indicate if evaluators of subjective components of study were masked to other aspects of the status of the participants |  |  |  |
| 6) Describe any assessments undertaken for quality assurance purposes (e.g., test/retest of primary outcome measurements) |  |  |  |
| 7) Explain any patient exclusions from analysis |  |  |  |
| 8) Describe how confounding was assessed and/or controlled. |  |  |  |
| 9) If applicable, explain how missing data were handled in the analysis |  |  |  |
| 10) Summarize patient response rates and completeness of data collection |  |  |  |
| 11) Clarify what follow-up, if any, was expected and the percentage of patients for which incomplete data or follow-up was obtained |  |  |  |
