# Supplementary material for: A systematic review and meta-analysis of indoor bioaerosols in hospitals: The influence of heating, ventilation, and air conditioning
Source: PLoS One. 2021 Dec 23;16(12):e0259996. doi: 10.1371/journal.pone.0259996 (PMC8699671; doi:10.1371/journal.pone.0259996)
Supplement: S1 Table — (DOCX) [file pone.0259996.s003.docx]

The formulas used for conversion


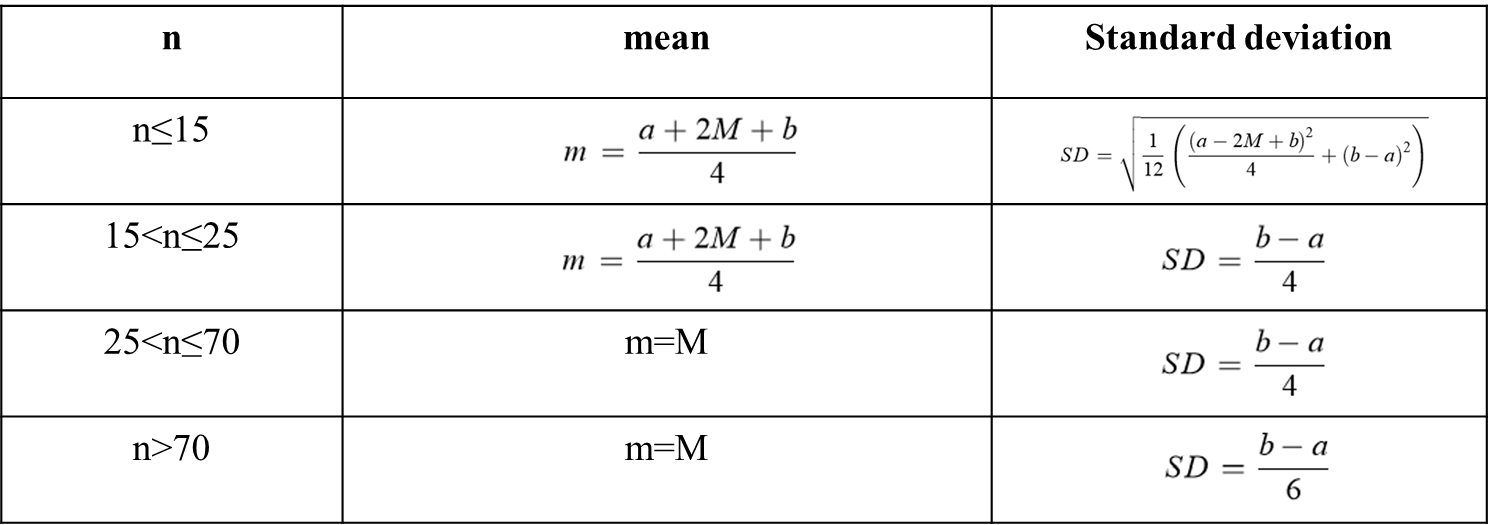


n, number of samples; m, mean; M, median; SD, standard deviation; a, minimum; b, maximum.
