# Supplementary material for: A systematic review and meta-analysis of indoor bioaerosols in hospitals: The influence of heating, ventilation, and air conditioning
Source: PLoS One. 2021 Dec 23;16(12):e0259996. doi: 10.1371/journal.pone.0259996 (PMC8699671; doi:10.1371/journal.pone.0259996)
Supplement: Attachment — Submitted filename: Response to Reviewers.docx [file pone.0259996.s006.docx]

**Response to Reviewer #1**

1. Comment: I suggest you include that HVACs are for maintaining good indoor air quality. There is often no real ventilation strategy dedicated to usersand the activities carried out. Ventilation must be part of an organic strategy and measures.

1. Reply: We believe that the HVAC systems are no doubt for maintaining good indoor air quality and the HEPA filters can also help. (Page 29, line 412-415).

2.Comment: In the discussion read and quote the articles.

Gola m. capolongo S. et al.

Indoor air in healing environments: Monitoring chemical pollution in inpatient rooms. Facilities, 2019, 37(9-10), pp. 600–623.

2. Reply: Thank you very much for your recommendation. These articles on chemical pollution and energy efficiency complement our research. This article has been quoted on page 31, lines 455-456.

3. Comment: Existing guidelines for indoor air quality: The case study of hospital environments

SpringerBriefs in Public Health, 2017, (9783319491592), pp. 13–26

3. Reply: Thank you very much for your recommendation. This article has been quoted on page 3, lines 62-64

4. Comment: Indoor Air Quality in Inpatient Environments: A Systematic Review on Factors that Influence Chemical Pollution in Inpatient Wards

Journal of Healthcare Engineering, 2019, 2019, 8358306.

4. Reply: Thank you very much for your recommendation. This article has been quoted on page 31, lines 466-467.

5. Comment: The Dichotomy between Indoor Air Quality and Energy Efficiency in Light of the Onset of the COVID-19 Pandemic

Atmosphere 2021, 12, 791. https://doi.org/10.3390/atmos12060791

5. Reply: Thank you very much for your recommendation. This article has been quoted on page 32, lines 478-479.

Thank you again for your positive comments and valuable suggestions to improve the quality of our manuscript.

**Response to Reviewer #2**

1. Comment: Some paragraphs were founded on critical grammar errors.

1. Reply: Thank you very much for pointing out our problem. We have tried our best to improve the manuscript and made some modification to the manuscript. These changes will not influence the content and framework of the paper. And here we did not list the changes but marked in red in file labeled 'Revised Manuscript with Track Changes'. We appreciate for your warm work earnestly and hope that the correction will meet with approval.

We have made changes to the syntax of many sentences, for example:

Original: Databases of Embase, PubMed, Cochrane Library, MEDLINE, and Web of Science were searched from 1st January 2000 to 31th December 2020.

Revised: Databases of Embase, PubMed, Cochrane Library, MEDLINE, and Web of Science were searched from 1st January 2000 to 31st December 2020 (Page 1, line 17-18).

Original: However, the coronavirus disease 2019 (COVID-19) pandemic has raised people’s concern that HVAC systems may increase the risk of airborne diseases if they are not well designed or managed properly.

Revised: However, the coronavirus disease 2019 (COVID-19) pandemic has raised concerns that HVAC systems may increase the risk of airborne diseases if not well designed or properly managed (Page 3, line 62-64).

Original: After conversions, there were still multiple data with consistent classification, for example, some articles carried out the comparison between working state and non-working state [28] or the comparison among different kinds of operating [29].

Revised: After the conversions, there were still multiple data with consistent classification. For example, some articles compared working and non-working states [28] or compared different medical operation procedures [29] (Page 7, line 152-154).

Original: In the conditions using HEPA filters (bacterial count: 36.90 ± 40.06 CFU/m3; fungal count: 9.46 ± 9.63 CFU/m3), lower mean bioaerosol concentrations were obtained than in the conditions using other filters (bacterial count: 57.83 ± 85.06 CFU/m3; fungal count: 12.21 ± 14.22 CFU/m3).

Revised: Conditions wherein HEPA filters were used (bacterial count: 36.90 ± 40.06 CFU/m3; fungal count: 9.46 ± 9.63 CFU/m3) showed lower mean bioaerosol concentrations than those wherein other filters were used (bacterial count: 57.83 ± 85.06 CFU/m3; fungal count: 12.21 ± 14.22 CFU/m3) (Page 19-20, line 255-258).

Original: We usually think that in restricted areas of hospitals, the concentration of microbial bioaerosols should be lower because of more stringent management and disinfection measures.

Revised: It is typically thought that the concentration of microbial bioaerosols should be lower in restricted areas of hospitals because of more stringent management and disinfection measures (Page 25, line 326-327).

Original: These four studies are part of the difference in the sampling area in the operating room.

Revised: These four studies provide information on the differences in the sample areas of operating rooms (Page 28, line 386-387).

2. Comment: Therefore, need to proofread one native researcher and provide the proofreading certificate supplementary file, and sent the revised version for peer review.

2. Reply: In the revised manuscript we have employed an English-language editing service, *Editage* to polish our wording. And a language certificate supplementary file has been uploaded (*Certificate_of_editing-BSEQN_1_5_l3xzfkt2-g.pdf*). We hope that the polished manuscript meets the publication requirements. We would like to express our great appreciation to you for your efforts spent on our manuscript.
